# Editorial: Convolutional neural networks and deep learning for crop improvement and production

**DOI:** 10.3389/fpls.2022.1079148

**Published:** 2022-11-18

**Authors:** Wanneng Yang, Gregorio Egea, Kioumars Ghamkhar

**Affiliations:** ^1^ National Key Laboratory of Crop Genetic Improvement, National Center of Plant Gene Research, Hubei Hongshan Laboratory, Huazhong Agricultural University, Wuhan, China; ^2^ Area of Agroforestry Engineering, School of Agricultural Engineering, University of Seville, Seville, Spain; ^3^ Margot Forde Germplasm Centre, Grasslands Research Centre, AgResearch, Palmerston North, New Zealand

**Keywords:** convolutional neural networks (CNN), deep learning (DL), big data, pattern recognition, object recognition, crop improvement, crop production, artificial intelligence

With the development of high-throughput phenotyping (HTP) technology, the large amount of phenotypic data has provided the breeders with new opportunities of accurate and repeatable phenotyping or phenomics (Ghamkhar et al., 2019; Roitsch et al., 2019; Yang et al., 2020). There is also the question of what to do with the increasingly substantial amounts of generated data using these technologies. Traditionally, we have used manual/visual methods to estimate or measure plant phenotype. These traditional methods are time-consuming and labour intensive hence the need to technological innovation in phenotypic technologies (Furbank and Tester, 2011; Ubbens and Stavness, 2017). Nowadays, with the advent of image processing enabling software handling large amount of data is more manageable. For phenotyping platforms of industrial scale in controlled environments, a simple background, a controlled environment, and a streamlined image processing method make it possible to fully automate high-throughput phenotypic data acquisition and analysis. However, for complex working conditions, such as field and phenotyping platforms with complex backgrounds, the challenges of image processing increase dramatically. Further, the robustness of the program will also decline due to the challenges of repeatability, which inadvertently will increase the costs of programming and the labour cost for manual intervention. In more complex cases, such as segmentation of specific parts of a plant, image processing methods are more challenging to achieve congruent results due to the complexity of features. Recent advances in deep learning technologies will ease overcoming this bottleneck.

The purpose of object detection is to find out the location of an object in the image and classify it. This is a combination of object localization and image classification. Compared with image classification, where the classification can only get an image of the subject, the object detection task is used to detect the image of a number of different categories of individuals, often used to count or target tasks, and is widely used in automated tasks, so as to realize the recognition of pedestrians, vehicles and traffic light detection (Xiao et al., 2020).

## Species identification and discrimination

The application of deep learning technology in phenotyping is mostly in image processing. The core process of image classification task is to assign labels to the images of interest (Bateman et al., 2020; Chen et al., 2021). The use of deep learning network can classify images end-to-end, without the need to extract the features of the target and quantify them into data as in traditional image processing methods. Large-scale data acquisition using UAVs are examples for using deep learning in order to decimate the data processing time. Zhang et al. demonstrate the use of UAV and CNN to identify and map weeds in various areas of the field, which can effectively help the more efficient control and removal of weeds. Application programming interface (API) implementation of the PyTorch deep learning library has been used in this study with a range of precision depending on the weed species and type. Not surprisingly, the authors suggest that more than one model would be needed to improve the weed mapping involving more than one species. Fujiwara et al. applied convolutional neural network on UAV data and quickly classified and segmented grasses in UAV images, thereby quantifying the coverage legumes in the area of interest, effectively achieving the appropriate management of a grass and legume mixture. Yue et al. have applied deep learning as well as partial two pattern recognition models (least squares discriminant analysis (PLS-DA) and support vector machine (SVM)) to identify the medicinal plant *Paris polyphylla* var. *yunnanensis* using spectroscopy data. Their results show that the deep learning model had clear advantage in the identification of this plant. The direct use of two-dimensional correlation spectroscopy (2DCOS) shows the strength of deep learning for multi-class image data.

## Crop disease recognition

Convolutional neural network (CNN) can effectively identify plant disease categories that would have only been possible by the experts in the past. Wang et al. use a deep learning model called Coordinated Attention EfficientNet (CA-ENet) to identify different apple diseases. Their method’s accuracy reached 98.92%, and the average F1-score reached 0.988, which is superior to many mainstream models and has a certain robustness. Their model learnt both the channel and spatial location information of important features. The targeted design network can better realize the purpose of agricultural application. For example, the proposed deployment based on a dilated convolution capsule network (DCCapsNet), proposed by Xu et al., can quickly capture and define diseased apple leaves, and potentially enable early prevention of apple diseases. Deep learning object detection has obvious advantages in counting, positioning, and judgment, which is a milestone that is difficult to achieve by traditional image processing methods. Zhou et al. used deep learning image classification technology to identify rice diseases. When different diseases cause similar or the same symptoms, simultaneous training is better than separate training. When the symptoms are significantly different, any method can achieve high accuracy.

## Reproductive yield measurement

The detection model to identify grains in the rice panicle and whether the grain is full or bare is used by Guo et al., in order to define rice seed setting rate (RSSR) more accurately and measure reproductive yield in a high-throughput manner. In the study of plant phenotype, object detection task has also been very widely used. In general, the object detection task in plant phenotype is to find and define the regions of great significance in the plant, specifically for breeding purposes. Zang et al. use the improved classic YOLOv5s detection model, by introducing an efficient channel attention module (ECA), to identify wheat spikes with a detection accuracy of 71.61%, allowing for rapid and accurate wheat reproductive yield estimation. This method is specifically useful in complex field environments. New methods and new ideas beyond deep learning are also emerging. Ensemble learning, for example (Shahhosseini et al.), predicts grain yield directly from images and some environmental data. Different from mature deep learning application schemes such as network application and modification network, how to mine new applications of deep learning in phenotyping is an important part of the future developments.

## Identification of different stages of growth

The use of data collected by UAVs helped effective identification of the growth stages of rice seedlings (Tan et al.), thereby providing valuable time-sensitive advice for cultivation management. This is an alternative high-throughput method to the current labor intensive and subjective manual measurement practice. Histograms of oriented gradients (HOGs) were combined with the support vector machine (SVM) classifier to recognize and classify three growth stages.

## Segmentation for morphometrics of micro and macro organs

Compared with the image segmentation based on the traditional image processing technology, image segmentation based on deep learning techniques can handle different scales in the target segmentation task and has the ability to solve the problem of complicated background, therefore it has great application prospect in agriculture (Ghosh et al., 2019). The use of RGB, near-infrared images or a combination of both has been shown to be accurate in seed quality assessment (Hansen et al., 2016). In this issue, Wang et al. combine these two imaging modalities and the watershed algorithm to segment corn seeds and then use deep learning to identify seed defect. The authors report an accuracy of >95%. From macro to micro, deep learning image segmentation technology can also be applied to the segmentation of microscopic images such as stomata (Gibbs et al.), which can realize fully automatic morphological measurement of stomata and maximal conductance estimation of stomata. As this study shows, deep learning image segmentation technology can extract specific targets at the pixel level, and the information obtained is larger, but the drawback is that the difficulty of data labeling is also greatly increased.

Segmentation of wheat leaves under outdoor conditions is a challenging task, but it is also a prerequisite for high-throughput field phenotype. The classical semantic segmentation model DeepLab V3 can effectively segment wheat leaves under complex field background with a mIOU of 0.77, which lays a foundation for quantifying canopy cover and deriving traits in the field (Zenkl et al.). Similarly, by deploying an improved fully convolutional network with channel and spatial attention on an intelligent harvesting robot, the branches and fruits of guava trees have been segmented in real time to plan collision-free paths for fruit picking (Lin et al.).

In pixel-level image segmentation, extracting the image from the area of interest is an important and difficult challenge in automatic image processing. Nowadays, in the application of deep learning in plant phenotyping, data are generally collected by researchers themselves, and the difficulty of data acquisition and data labeling is self-evident. Apart from the industry’s data, there are a large number of public data sets, and transfer applications in industry only need to conduct small transfer learning on pre-trained models to obtain reasonable results. Unfortunately, few phenotype-related data are available in publicly available datasets, which makes it more important to develop large-scale phenotype-specific datasets and pre-trained models, which can greatly reduce the input of data acquisition for researchers, such as AgriNet’s pioneering work (Al Sahili and Awad). Consistent with the computer industry, actively adopting new technologies, adapting measures to local conditions, and expanding innovation may make deep learning technology play even more a more significant role in future phenotyping.

## Author contributions

WY initially drafted the manuscript. All authors listed have made substantial, direct, and intellectual contribution to the work. KG finalized and submitted the manuscript. All authors have approved the final manuscript for publication

## Funding

This work was supported by grants from the National Natural Science Foundation of China (U21A20205), Key projects of Natural Science Foundation of Hubei Province (2021CFA059), and Fundamental Research Funds for the Central Universities (2021ZKPY006), HZAU-AGIS Cooperation Fund (SZYJY2022014) for WY. New Zealand’s Ministry of Business, Innovation and Employment (Project number C10X1701) and AgResearch Ltd provided financial support for KG.

## Acknowledgments

We thank all authors and reviewers for their contributions to this Research Topic and for the support of the editorial office.

## Conflict of interest

The authors declare that the research was conducted in the absence of any commercial or financial relationships that could be construed as a potential conflict of interest.

## Publisher’s note

All claims expressed in this article are solely those of the authors and do not necessarily represent those of their affiliated organizations, or those of the publisher, the editors and the reviewers. Any product that may be evaluated in this article, or claim that may be made by its manufacturer, is not guaranteed or endorsed by the publisher.
